# Books and Magazines

**Published:** 1908-02

**Authors:** 


### Books and Magazines.

SQUIBBS' MATERIA MEDICA—PRICE LIST, 1908.—A Complete Alphabetical List of the Squibbs' Products, embracing the articles in the U. S. Pharmacopeia (VIII Revision) and the National Formulary, together with the non-official Chemicals, Pharmaceuticals, Tablets and Newer Remedies in general use; setting forth their origin; Latin and English titles; synonyms; physical and chemical characteristics; incompatibles; antidotes; therapeutic indications; doses, etc. E. R. Squibbs & Sons, Publishers, manufacturing chemists to the medical profession since 1858. New York and Brooklyn.

Practitioners of medicine will find this book very useful as a *vade mecum.* It is an epitome of the dispensatory. The name of

Squibbs stands for purity and reliability. The firm will be glad to answer any legitimate inquiry about anything connected with their specialty. Mention the "Red Back."

---

THE INTERNAL SECRETIONS AND THE PRINCIPLES OF MEDICINE. By Chas. E. de M. Sajous, M. D., Fellow of the College of Physicians of Philadelphia; member of the American Philosophical Society, the Academy of National Sciences of Philadelphia, Knight of the Legion of Honor, and Officer of the Academy of France; Knight of the Order of Leopold of Belgium, etc.; formerly Lecturer on Laryngology in Jefferson Medical College, and Professor of Laryngology and Dean of the Faculty in the Medico-Chirurgical College; formerly Professor of Anatomy and Physiology in the Wagner Institute of Science. Vol. 1, with 42 illustrations. Philadelphia: F. A. Davis Co., Publishers. Cloth, $6.50.

Vol. 2 has since appeared, and had attention in our December number. The work consists of the two volumes; $6.50 per volume.

This is by far the most important as well as the most interesting medical work that has appeared within a half century. I make no exceptions. It will revolutionize the practice and upset all previous doctrines as to physiological chemistry.

The Commoner Diseases of the Eye—How to Detect and How to Treat Them.—For Students of Medicine. 280 illustrations (many original) and 8 colored plates. By Casey A. Wood, M. D., C. M. D. C. L., Professor of Ophthalmology, Northwestern University, Chicago; Ophthalmic Surgeon to St. Luke's Hospital and Wesley Hospital, Chicago; Consulting Ophthalmologist to Cook County Hospital and Passavant Memorial Hospital; ex-Chairman of the Ophthalmic Section, American Medical Association; formerly President of the American Academy of Medicine, and of the American Academy of Ophthalmology and Otolaryngology; Mitglied der Ophthalmologischen Gesellschaft, etc.; and Thomas A. Woodruff, M. D., C. M., L. R. C. P. (London), Ophthalmic Surgeon, St. Luke's Hospital, and St. Anthony de Padua Hospital, Chicago; member Ophthalmic Section American Medical Association; Fellow of American Academy of Medicine; Fellow of American Academy of Ophthalmology and Otolaryngology; Mitglied der Ophthalmologischen Gesellschaft, etc. Third edition, enlarged and improved, with index. Chicago: W. T. Keener & Co., 1907.

This book has been before the profession several years, and has become a popular favorite. The present volume is the third edition and is greatly improved. No one can dispute the high standing of its authors; and its aim and purpose are set forth in the title. It "considers ophthalmology from the standpoint of the physician in general practice," and not alone from that of the expert specialist.

---

Light and X-Ray Treatment of Skin Diseases. By Malcolm Morris, F. R. C. S. (Ed.), Dermatologist to King Edward the Seventh's Hospital for Officers; Surgeon to the Skin Department of the Seaman's Hospital; Consulting Surgeon to the Skin Department of St. Mary's Hospital; and S. Ernest Dore, M. D., Cantrol Assistant in the Skin Department of the Middlesex Hospital. With 12 plates. Chicago. 1907. W. T. Keener & Co. Price, $1.50, net.

In the introductory the author says: "This book is not intended to be an exhaustive monograph on the subject of which it treats, but a concise summary of the methods of application and results of Finsen's light treatment, X-rays, and other therapeutic agencies which have been introduced into dermatological practice within the last ten or twelve years. The conclusions are based mainly on our own experience; but the work of others in the same field has received due attention. Our aim has been not to give records of 'cures,' but to set forth in their true light the facts we have observed, and to help the reader to form an accurate estimate of the value of the several methods described."

As such, the physician who is called to treat skin diseases will find this book of value and assistance.

TREATMENT OF CEREBRO-SPINAL MENINGITIS BY THE SUBCUTANEOUS INJECTION OF THE FLUID OBTAINED BY LUMBAR PUNCTURE.—Radman (*Semaine médicale*, No. 28) reports two successful cases of the injection of cerebro-spinal fluid obtained from the patient himself by lumbar puncture in the treatment of cerebro-spinal fever. He found the meningococcus in the fluid, of which 35 c.c. were drawn from the spinal canal, and 8 c.c. were immediately injected under the skin of the arm. There was no local reaction. Three days later the temperature had fallen, and at the end of fifteen days all the morbid symptoms had disappeared. The object of the treatment is to provoke a curative reaction.

